# Characterizing the Binding Sites for GK Domain of DLG1 and DLG4 via Molecular Dynamics Simulation

**DOI:** 10.3389/fmolb.2020.00001

**Published:** 2020-01-23

**Authors:** Hongwei Li, Qiong Chen, Changyu Shan, Chunling Guo, Xiuming Yang, Yingchun Chen, Jinwei Zhu, Qin Ouyang

**Affiliations:** ^1^Department of Pharmaceutical Chemistry, Third Military Medical University, Chongqing, China; ^2^Department of Neurology, Xinqiao Hospital, Third Military Medical University, Chongqing, China; ^3^Bio-X Institutes, Key Laboratory for the Genetics of Developmental and Neuropsychiatric Disorders, Ministry of Education, Shanghai Jiao Tong University, Shanghai, China

**Keywords:** DLG, GK domain, molecular dynamics simulation, free energy calculation, molecular-mechanics-generalized-born-surface-area

## Abstract

Discs-large (DLG) is a member that belongs to the membrane-associated guanylate kinase (MAGUK) family. The GK domain of DLGs has evolved into a protein–protein interaction module that could bind with kinds of proteins to regulate diverse cellular functions. Previous reports have demonstrated the GK domain of DLGs functioned as a phosphor-peptide-binding module by resolving the crystal structures. Here we investigated into the interactions of DLG1 and DLG4 with their reported phosphor-peptides by molecular dynamics simulations. Post-dynamics analysis showed that DLG1/4 formed extensive interactions with phosphorylated ligands, including hydrophobic and hydrogen bonding interactions. Among them, the highly conserved residues among the DLGs in phosphor-site and β5 sheet were crucial for the binding according to the energy decomposition calculations. Additionally, the binding interactions between DLG4 and reported unphosphorylated peptides including MAP1A and designed GK inhibitory (GKI-QSF) peptides were analyzed. We found the key residues that played important roles in DLG4/unphosphorylated peptide systems were very similar as in DLG4/phosphor-peptide systems. Moreover, the molecular dynamic simulation for the complex of DLG1 and GKI-QSF was carried out and predicted that the GKI-QSF could bind with DLG1 with similar *K*d value compared to DLG4/GKI-QSF, which was verified by using ITC assay (*K*d = 1.20 ± 0.29 μM). Our study might be helpful for the better understanding of the structural and biological function of DLGs GK domain and encourage the discovery of new binders.

## Introduction

The membrane-associated guanylate kinase (MAGUK) family is a widely expressed and well-conserved group of scaffold proteins, providing a structural framework for protein complexes formation and playing an essential role in the regulation of cellular signal transduction (Kim, 1995; Funke et al., 2005). Guanylate kinase (GK) domain, originally served as catalyst for the reversible phosphate transfer from ATP to GMP (Anderson, 2015), containing a binding site for the GMP. However, the GK domain of MAGUK which had evolved into a protein–protein interaction module without any detectable catalytic activity, could bind with kinds of proteins. These GK domain binding proteins play diverse roles in tissue development, cell polarity control, synaptic formation and plasticity (Zhu et al., 2012).

Discs large (DLG) family scaffold proteins, a class of MAGUKs, include five paralogs in mammals (DLG1–5) via two rounds of genome duplications throughout the evolution (Wakabayashi et al., 2003; Roberts et al., 2012). DLGs are known as synapse-associated protein SAP97 (DLG1), PSD-93/Chapsyn-110 (DLG2), SAP102 (DLG3), PSD-95/SAP90 (DLG4) and P-DLG (DLG5), respectively. DLGs employ similar function as key architectural proteins responsible for anchoring various postsynaptic components including glutamate receptors, downstream scaffold proteins and signaling enzymes (Zhu et al., 2016a). As a component of the scribble polarity complex, DLGs also play critical roles in diverse cellular processes including the regulation of apical-basal polarity of epithelial cells (Wang and Margolis, 2007), as well as other polarity processes such as asymmetric cell division and cell invasion. Interfering the function of DLG leads to uncontrolled epithelial cell proliferation and epithelial-to-mesenchymal transition (Roberts et al., 2012; Liu et al., 2019; Marziali et al., 2019).

Undoubtedly, all five DLGs share a high-amino-acid sequence identity and a very similar modular structural organization, which consist of three PDZ domains at the N-terminus and a SH3–GK tandem at the C-terminus half (Craven and Bredt, 1998). Similarly, the DLGs GK domain folds into similar structurally supramodules and shares unique target recognition mode with their binding partners, such as LGN, GKAP/DLGAP/SAPAP, SPAR, AKAP79, MAP1A, GAKIN, BEGAIN, and GUK-holder (Zhu et al., 2012). Recently, seven crystal structures of DLG with different peptides were reported, including DLG1 GK/p-LGN (PDB ID: 3UAT), DLG4 GK/p-LGL2 (PDB ID: 3WP0, 3WP1), DLG4 GK/p-SAPAP (PDB ID: 5YPO), DLG4 GK/Kif13b (PDB ID: 5B64), DLG4 GK/MAP1A (PDB ID: 5GNV), and DLG4 GK/GKI-QSF (PDB ID: 5YPR) (Table S3). The structural biology revealed that these complexes shared several highly conserved interactions, including the phosphor-site, formed by Arg755, Tyr796, and Glu761 in DLG1 (Arg568, Tyr609, and Glu574 in DLG4), and hydrophobic site formed by Tyr791, Tyr796, and Gly 789 in DLG1 (Tyr604, Tyr 609, and Gly602 in DLG4) (Zhu et al., 2011, 2014, 2016b, 2017; Xia et al., 2017). However, the dynamic interactions of DLGs and phosphorylated/unphosphorylated peptides, which should be helpful for understanding their physiological function (Hu et al., 2019; Luo et al., 2019), have not been fully derived. In this paper, we investigated into the structural similarities and differences of the GMP-binding subdomain of DLG1 and DLG4 by using molecular dynamic simulation of reported crystal structures in order to understand the properties of GK domains and pursue the development of new binder.

## Methods

### Preparation of Protein Crystal Structures

The crystal structures were obtained from RCSB Protein Data Bank (PDB ID: 3UAT, 3WP0, 5YPO, 5GNV, and 5YPR) (Zhu et al., 2011, 2014, 2017; Xia et al., 2017). In order to improve the efficiency of the simulation, the excess part of the DLG1 crystal structure at the N-terminus was deleted compared to the DLG4, because these amino acids were far from the binding pocket. Then, the structures were prepared by SYBYL-X 2.0 software (Tripos International, 2012) with the Powell method under AMBER7 FF99 force field and AMBER charges. Protonation states of ionizable residues and histidine residues were predicted according to the microenvironment and p*K*a values calculated by the PDB2PQR Server (http://nbcr-222.ucsd.edu/pdb2pqr_2.0.0/) (Dolinsky et al., 2004) at pH = 7.0.

### Molecular Docking Study of DLG1/GKI-QSF Complex

The crystal structures of DLG1 and GKI-QSF peptide were extracted from the DLG1/p-LGN and DLG4/GKI-QSF complex, respectively (PDB ID: 3UAT, 5YPR). For protein and protein docking, ZDOCK server (http://zdock.umassmed.edu/) was applied to generate the initial docking model (Pierce et al., 2014; Shan et al., 2019). Preparared structures of DLG1 and GKI-QSF peptide were uploaded to the web server. Docking was carried out with the default parameters. A reasonable complex was selected among predicted complexes, in which GKI-QSF located at the same site as DLG4.

### Molecular Dynamics Simulation

All MD simulations were carried out using AMBER14 (Case et al., 2014) with ff14SB force field (Maier et al., 2015). The structures were prepared with the *tleap* module (Case et al., 2005) and minimized with *pmemd.MPI*. The MD simulations were run with *pmemd.cuda.MPI* executable using Graphical Units Processors module (Götz et al., 2012; Salomon-Ferrer et al., 2013). The systems were neutralized with Na^+^ or Cl^−^ firstly, then solvated in the TIP3P water model (Jorgensen et al., 1983) and subsequently placed into a regular hexahedron box with a minimal distance of 12 Å for the solute from the box borders. The AMBER parameters of phosphorylated serine were obtained from AMBER parameter database (http://research.bmh.manchester.ac.uk/bryce/amber) (Craft and Legge, 2005). After minimization and equilibration, MD simulations for the different systems were performed, respectively. Three hundred and fifty nanoseconds of MD simulations were run under periodic boundary conditions using NPT ensemble at 300 K (Sun et al., 2018, 2019).

### Trajectory Analysis

The simulation trajectories were analyzed using the *cpptraj* module (Roe and Cheatham, 2013) of Amber 14. The root mean square deviation (RMSD), root mean square fluctuation (RMSF) and hydrogen bonds were calculated. The equilibrium of the system was determined according to RMSD values. From MD simulation times when the protein reached equilibrium, the average structures of the models were calculated using the *cpptraj* module. The mechanisms of binding between the DLG1/4 and phosphor-peptides were characterized using LigPlot+ (Laskowski and Swindells, 2011; Suresh et al., 2018).

### Calculation of Binding Free Energies

To calculate the binding free energies of DLG1 and DLG4 with their respective ligands, 350 ns MD simulations were performed using the aforesaid MD protocol, until the systems reached equilibrium. The binding free energies were calculated using the MM/GBSA method (Hou et al., 2011) implemented in AMBER 14. Totally 100 snapshots were extracted from the equilibrium trajectory for MM/GBSA free energy calculation. Per residue energy decomposition was also performed to evaluate the energy contribution of each residue in the systems. All the other parameters were kept as default value.

### Protein Expression and Purification

DLG1 GK domain was cloned into a modified pET-15b vector with N-terminal His6-tag. The construct was expressed in BL21 (DE3) Escherichia coli cells and induced by 0.2 M isopropyl-β-D-thiogalactoside (IPTG) for 18 h at 16°C. His6-tagged protein was first purified using the Ni^2+^-NTA agarose affinity chromatography (GE Healthcare), and then further purified by size-exclusion chromatography (Superdex-200 26/60, GE Healthcare) in the buffer containing 50 mM Tris pH 8.0, 100 mM NaCl, 1 mM EDTA and 1 mM DTT.

### ITC Assay

ITC measurements were carried out on a MicroCal-iTC200 system (Malvern) in a buffer containing 50 mM Tris pH 8.0, 100 mM NaCl, 1 mM EDTA, and 1 mM DTT at 25°C. The concentrations of proteins loaded into the syringe (GKI-QSF) and the cell (DLG1 GK) were 0.5 and 0.05 mM, respectively. The titration data were analyzed using Origin7.0 from MicalCal and fitted by a one-site binding model.

## Results

### The Comparison of GK Domain in DLGs

**Figure 1:**
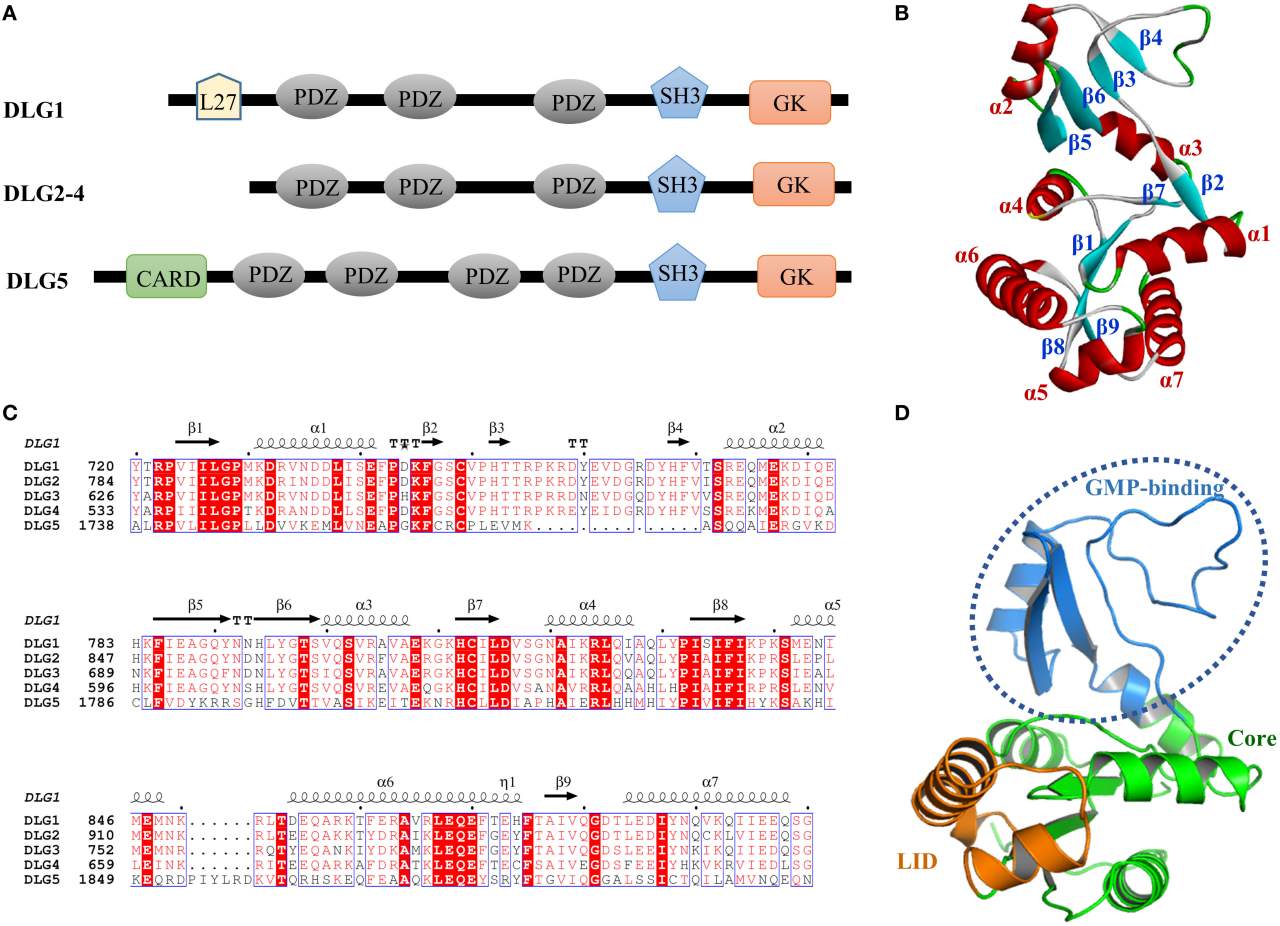
**(A)** Domain organization of the DLGs protein. **(B)** The amino acid sequences alignment of the DLGs GK domains using Clustal W. Highly conserved residues (conservation score > 0.7) were framed in blue according to physico-chemical properties. **(C)** Structural features of the DLG1 GK domain. **(D)** The subdomain of the DLG1 GK domain. The GMP-binding subdomain, Core subdomain, and LID subdomain were colored marine, green, and orange, respectively.

The human DLGs protein contain the PSD95/DLG/ZO-1 (PDZ) domain, the Src homology3 (SH3) interaction module, the Lin-2/Lin-7 (L27) domain, the Caspase Recruitment domain (CARD) and the guanylate kinase (GK) domain. In detail, DLG2-4 have three PDZ domains, one SH3 domain and GK domain, while DLG1 has an extra L27 domain at the N-terminus and DLG5 has one CARD and one more PDZ domain at the N-terminus (Figure 1A).

In order to compare the amino acid sequence similarity among the GK domains of DLG1-5, we performed the amino acid sequences alignment using the Clustal W algorithm (Robert and Gouet, 2014; Madeira et al., 2019). As shown in Figure 1B, the GK domains in DLG1-5 showed more than 97% amino acid sequence consensus. In terms of sequence, GK domains of DLGs were similar to each other with the exception that DLG5 GK domain had two small segments with slightly different sequences. Overall, GK domain, which was a more conserved sequence within this protein family, might play a common biological function acting as a protein–protein binding module.

From the protein structure analysis, the GK domain consisted of 7 α-helices (α1-α7) and 9 β-sheets (β1-β9), forming the Core, LID and GMP-binding subdomain (Figures 1C,D). Previous reports had characterized the phosphorylated ligands interacted with the GMP-binding subdomain which was an ancestral enzymatic GKs used to coordinate the nucleotide phosphate (Zhu et al., 2012). As the crystal structures of DLG1 GK and DLG4 GK were available in the PDB database, we treated and aligned them, and found their three-dimensional structures were almost the same, while only a few loops showed a little bit of nuance. This indicated that the sequence and structure of the DLG GK domain were highly homologous, providing a structural basis for their similar properties to bind ligands in a phosphorylation-dependent manner.

### Molecular Dynamics Study of the Complex of DLG1 With P-LGN Peptide

To insight into the binding interaction of DLG1 and p-LGN peptide, we conducted a long time molecular dynamics simulation for 350 ns. The root mean square deviation (RMSD) was monitored during the simulation time to investigate the stability of DLG1/ p-LGN complex.

**Figure 2:**
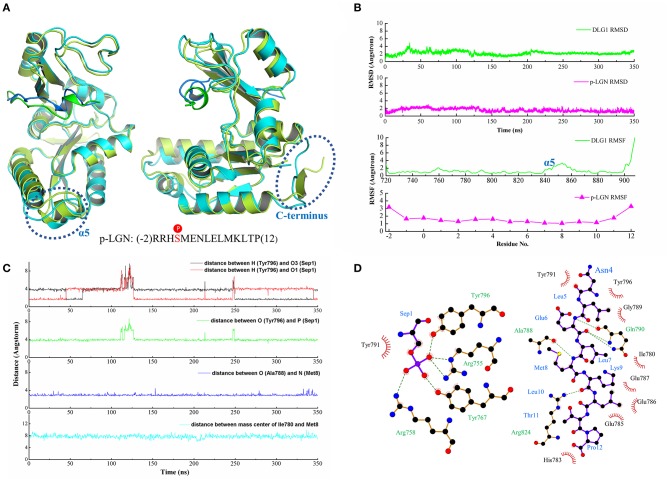
**(A)** The crystal structure superimposed on the last configuration after 350 ns of simulation for DLG1 and p-LGN. For the DLG1, the initial and the last configuration were shown in lemon and cyan, respectively. For the p-LGN, the initial and the last configuration were shown in the green and blue, respectively. **(B)** The RMSD and RMSF of DLG1 and p-LGN. **(C)** The representive dynamic distance within the key residues in DLG1/p-LGN complex. **(D)** The 2D visualization and interactions between DLG1 and p-LGN. The hydrogen bonds and the hydrophobic interactions were shown in green-dashed lines and red arcs, respectively.

Comparing with the crystal structure, the residues nearby the binding interface of DLG1 after molecular dynamics showed slight fluctuation, while loop at the C-terminus had great variation. The structure of p-LGN peptide also kept similar conformation, especially for the short α-helix (Figure 2A). From RMSD analysis, DLG1 and p-LGN reached stability at ~30 ns, with the RMSD value of 2.23 ± 0.45 and 1.57 ± 0.42 Å, respectively (Figure 2B). The RMSF analysis revealed the interaction of DLG1 with the ligand significantly reduced the mobility of the protein, since the residues of phosphor-site possessed low RMSF values <1 Å. The residues of LID subdomain (840–865) had larger RMSF with a highest value of 4 Å, which might result from the unstable loop structures. Similarly, the end of C-terminus (900–908) showed great variation with the RMSF values of 10 Å. On the other side, the RMSF of p-LGN peptide also had a lower deviation except the residues at both terminuses (residue at −2, 11, and 12) (Figure 2B). These results suggested the binding interface between DLG1 and p-LGN peptide was stable and suitable for the binding interaction analysis.

The MD simulation result showed that there were six important residues of DLG1 exhibiting strong interaction with p-LGN peptide by hydrogen bonds. We found the phosphate group of p-Ser (Sep) from the p-LGN could bind with Arg758, Arg755, Tyr767, and Tyr796 in DLG1, indicating the phosphorylation were very important for the binding. These residues in DLG1 formed a strong network of hydrogen bonds which were in a dynamic cyclical change with the phosphorylated serine of the ligand during the MD simulation (Figure 2C, Figure S1A). The distance between the heavy atoms (N or O atom) of the amino acid residues Arg758, Arg755, Tyr767, and Tyr796 of DLG1 and the phosphorus atom of Sep1 (p-LGN peptide) slightly vibrated within 3.60 ± 0.20, 3.79 ± 0.39, 3.70 ± 0.20, and 4.03 ± 0.53 Å, respectively. We also figured out some hydrogen bonds beside the phospho-binding site. For example, the distance between O atom of Ala788 (DLG1) and N atom of Met8 (p-LGN peptide) was kept at 2.93 ± 0.19 Å, as well as N atom of Gln790 (DLG1) and O atom of Glu6 (p-LGN peptide) was stable with value of 3.04 ± 0.20 Å (Figure 2C, Figure S1C). Beside hydrogen bonds, we also found some other binding interactions during the MD simulation, such as the hydrophobic interactions between Ile780 at α2 helix, Phe785, Ile786, Glu787, Ala788, Gly789, and Tyr791 at β5 sheet of DLG1 and p-LGN peptide (Figure 2D, Figure S1B). Interestingly, some binding interactions that were not obvious in the crystal structures were found by analyzing the MD simulation, such as the hydrogen bonds between Asp736, Asp766, Asp816 with Arg (-1) as well as Asp736, Asp766 with Arg (-2). In brief, besides Arg758, Arg755, Tyr767, and Tyr796 as the phosphate group binding sites, Asp736, Asp766, Asp816 and the hydrophobic residues of β5 sheet (788-791) for the binding interaction of p-LGN peptide were also suggested.

### Molecular Dynamics Study of the Complex of DLG4 With P-LGL2 Peptide

**Figure 3:**
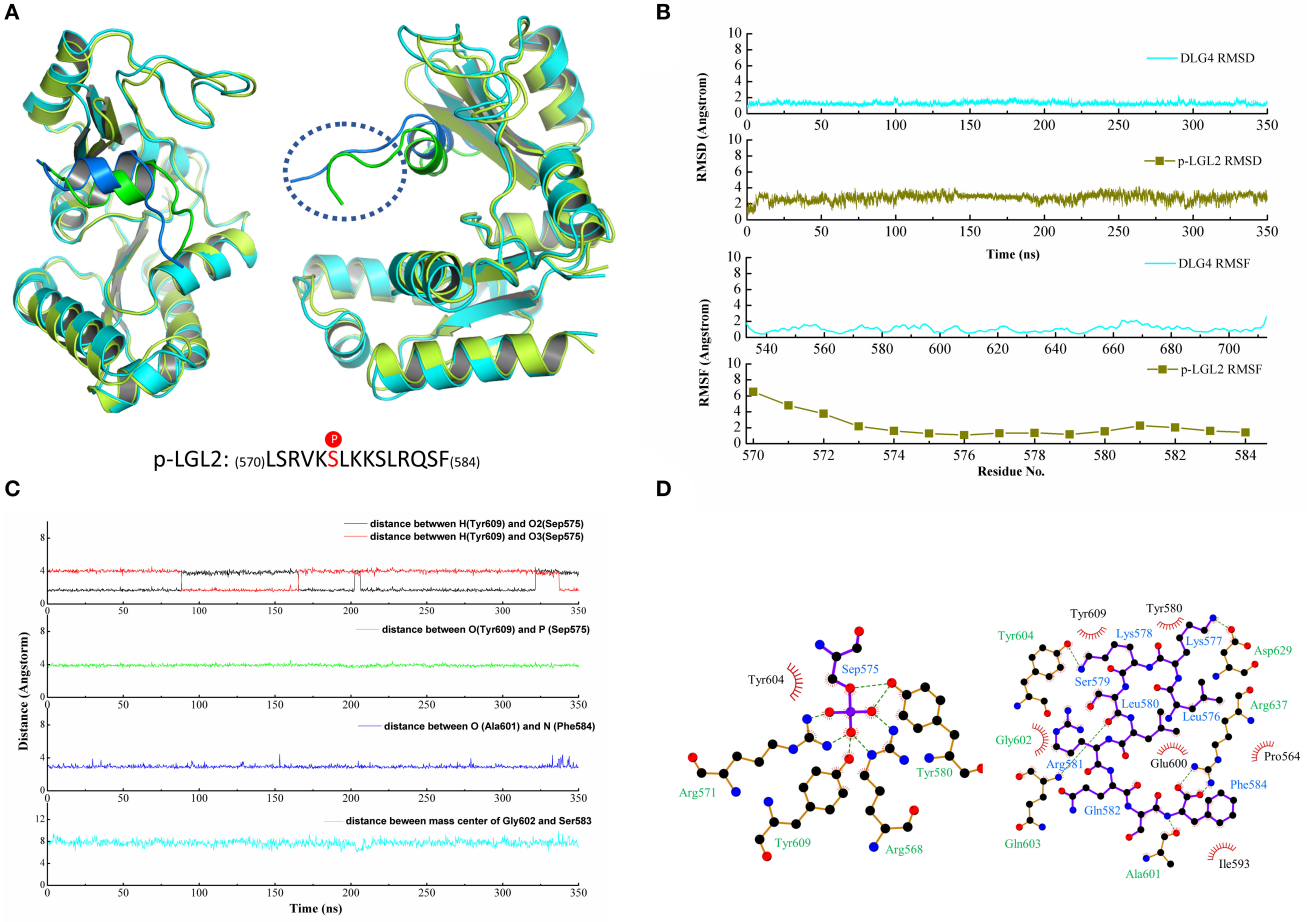
**(A)** The crystal structure superimposed on the last configuration after 350 ns of simulation for DLG4 and p-LGL2. For the DLG4, the initial and the last configuration were shown in limon and cyans, respectively. For the p-LGL2, the initial and the last configuration were shown in the green and blue, respectively. **(B)** The RMSD and RMSF of DLG4 and p-LGL2. **(C)** The representive dynamic distance within the key residues in DLG4/p-LGL2 complex. **(D)** The 2D visualization and interactions between DLG4 and p-LGL2. The hydrogen bonds and the hydrophobic interaction were shown in green-dashed lines and red arcs, respectively.

Similarly, a long time MD simulation of the DLG4/p-LGL2 complex was carried out for 350 ns. The structure of DLG4 was stable during the MD simulation while the residues at the N-terminus of the p-LGL2 fluctuated slightly larger than other regions since the loops at the N-terminus extended beyond the protein cavity causing a significant positional shift (Figure 3A). DLG4 maintained a stable structure throughout the simulation, with a RMSD value of 1.29 ± 0.19 Å, while p-LGL2 reached equilibrium at 10 ns, with a RMSD value of 2.82 ± 0.43 Å. The bigger RMSD value of p-LGL2 might result from the volatile N-terminus (570–573), which have a higher RMSF value of 4–6 Å (Figure 3B).

Similar to the DLG1/p-LGN, the residues of the phospho-binding site formed hydrogen bonds with phosphates group of p-LGL2 during MD simulation, such as Arg568, Arg571, Tyr580, and Tyr609, though we did not observe hydrogen bonds between Arg571 and p-LGL2 in the crystal structure. Arg637 at α4 helix was found to form the hydrogen bonds with Phe584, which stabilized the C-terminus of the p-LGL2. Asp629 at α1 helix was binding to Lys577 (p-LGL2) by hydrogen bonds, within the distance of 3.44 ± 1.10 Å. In addition, β5 sheet of DLG4 (601–604) was also found as important subdomain for the binding by hydrogen bonds and hydrophobic interactions, such as hydrogen bonds between Ala601 (DLG4) and Phe584 (p-LGL2) with distance of the heavy atoms (N or O) with values of 3.29 ± 0.65 Å. The residues including Pro564 at β3 sheet, Glu600, Ala601, Gly602, Tyr604 at β5 sheet, Ile593 at α2 helix and Arg637 at α4 helix formed the hydrophobic environment that contacted with the residues of p-LGL2. The distance vibration of the key residues between DLG4 and p-LGL2 was figure out in Figure 3C and other binding interactions could find in Figure 3D and Figure S2.

### The Important Binding Residues in DLG1 and DLG4

To explore the interactions in the two systems, MM/GBSA free energy calculation was performed. The binding free energy included VDW energy (ΔE_vdw_), electrostatic energy (Δ_Eelectrostatic_), the electrostatic contribution solvation free energy (ΔG_GB_) and the nonpolar solvation free energy (ΔG_SA_) with the values of −65.33 ± 6.65, −532.78 ± 81.94, 520.96 ± 76.35, and −11.62 ± 1.02 kcal/mol in DLG1 system and −52.85 ± 5.82, −490.41 ± 95.23, 472.51 ± 94.14, and −9.28 ± 0.71 kcal/mol in DLG4 system, respectively. According to the results, the ΔE_vdw_ and ΔE_electrostatic_ terms contributed mostly to the binding, although a large portion of ΔE_electrostatic_ was counteracted by the ΔG_GB_ terms, indicating that the interactions were primarily mediated by VDW interactions.

**Figure 4:**
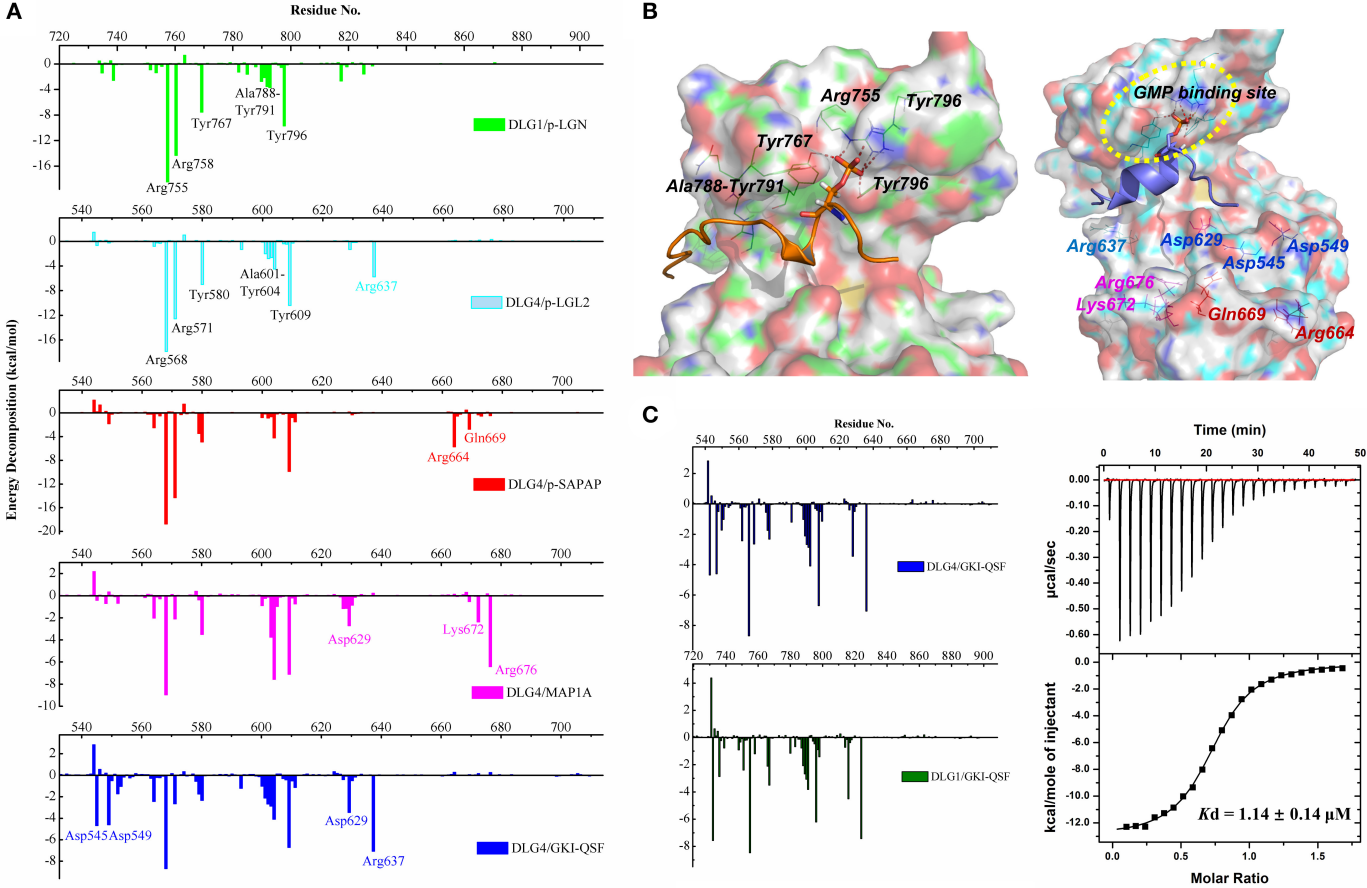
**(A)** Binding free energy decomposition of DLG1 and DLG4 systems. **(B)** The key residues for the interactions in DLG1/p-LGN (left) and DLG4/p-LGL2 (right) systems during the MD simulations. For the interactions of DLG4 with SAPAP, MAP1A, and GKI-QSF, specific amino acid residues in energy decomposition were marked with red, magenta and blue, respectively. **(C)** The binding free energy decomposition of DLG4/GKI-QSF and DLG1/GKI-QSF (left). The ITC-based measurement of the binding affinity of DLG1/GKI-QSF interactions (right).

Comparing the energy decomposition between DLG1 and DLG4 systems, the types of residues with energy contributions <1 kcal/mol were same, and the values of energy contribution were also similar. Two tyrosine and two arginine residues of the phospho-binding site contributed the most favorable energy, followed by the residues at the β5 sheet providing the important hydrophobic forces (Figure 4A). The detailed energy decomposition results of DLG1 and DLG4 systems could be found in Tables S1, S2. Four conserved residues Asp732, Asp736, Pro751, and His783 in DLG1 formed hydrogen bonds and hydrophobic interactions with p-LGN ligand, which was not observed in the DLG4/p-LGL2 complex (Figure 4B). We assumed the distinct amino acid sequences between p-LGL2 and p-LGN, rather than DLGs, might be the main reason for the difference of binding interaction. These results also indicated that the GK domains of DLG proteins might have the same binding pattern of the phosphorylated ligands due to the high sequence consensus and structural similarity.

### The Binding Interaction of Unphosphorylated Peptides

To further confirm the importance of the amino acid residues at GMP-binding subdomain, the molecular dynamic of the complex of DLG4 and p-SAPAP was also carried out (Figure S3A). The results of energy decomposition showed that the residues of the phospho-binding site and β5 sheet had similar energy contributions compared to DLG4/p-LGL2 (Figure 4A). It implied that the binding residues for kinds of phosphorylated target proteins did not have obvious difference, except the contribution from the unconservative residues far away from the protein cavity.

To generate more recognition mode of the DLG4 GK domain, the binding interactions of unphosphorylated peptides (MAP1A and GKI-QSF) were also analyzed by using energy decomposition strategy (Figures S3B,C). The results revealed that, just like the energy decomposition contribution profile of DLG4/p-LGL2, the residues of the phospho-binding site (Arg568, Arg571, Tyr580, and Tyr609) and β5 sheet (Ala601, Gly602, Gln603, and Tyr604) provided a beneficial binding free energy contribution, even for the unphosphorylated peptides mimicking the phosphor-Ser in binding to DLG4 (Figure 4A). Because DLG1/p-LGN, DLG4/p-LGL2 and DLG4/GKI-QSF had similar energy decomposition landscape, we inferred GKI-QSF might did not have obvious selectivity among these DLGs, and could bind with DLG1.

To accurately predict the binding interaction, molecular dynamic of DLG1/GK1-QSF was carried out. As a result, we found that the binding of DLG1 and GKI-QSF peptide was very stable (Figure S3D). From the energy decomposition diagram, the residues of DLG1 and DLG4 showed similar energy distribution, suggesting the GKI-QSF might bind with DLG1 with similar binding affinity (Figure 4C). To further confirm this, the binding affinity of GKI-QSF with DLG1 was determined by using ITC with value of 1.20 ± 0.29 μM, which is consistent with our prediction, since the reported *K*d values for the binding affinity of DLG4/GKI-QSF was 1.14 ± 0.14 μM (Zhu et al., 2017).

## Discussion

Previous studies have firmly demonstrated that the GK domains of DLGs were functionally indispensable, though the underlying mechanisms were poorly understood. Recent studies on the structural biology of DLG GK-mediated complexes had shown that the GK domain of DLGs was a multifunctional protein-protein interaction module that performed its biological functions by binding to different targets in a phosphorylation-dependent pattern (Xia et al., 2017). However, studies on phosphorylation-dependent binding patterns and amino acid residues that played the key role in binding have not been fully elucidated.

To demonstrate phosphorylation-dependent molecular binding mechanisms of DLG GK domain, crystal structures of DLG1 and DLG4 proteins were selected for molecular dynamics simulation and post-dynamics analysis. Because we focused on the GK domains of the DLGs, all the redundant amino acids, such as the SH3 domain in DLG1/p-LGN complex (PDB ID: 3UAT), were deleted for the molecular dynamic. The molecular dynamic studies also suggested that deleting these redundant residues had a slight effect on the results (Figure S4). Initially, we performed a dynamic simulation of 350 ns duration to obtain a relatively stable equilibrium of the system. Then, the post-analyses were conducted to identify the dynamic characteristics and to understand how the ligand bound to the protein in detail. The RMSD and the per-residue RMSF analysis discovered that the fluctuations decreased in binding site formed by the GMP-binding subdomain, while that in other regions were slightly increased. Similarly, the DLG4 displayed almost the same dynamic characteristics. In addition, free energy decomposition analysis showed the residues of the phospho-binding site played the crucial roles and contributed most favorable energy for the binding. The results were consistent with experimental reports that mutations of these residues in GK totally disrupted the interaction between p-LGL2 and DLG4 GK (Zhu et al., 2014).

Moreover, the molecular dynamic simulation and the post-dynamics analysis of the complexes of DLG4 with another phosphorylated peptide (p-SAPAP) and unphosphorylated peptides (MAP1A and GKI-QSF) were also implemented to further confirm the importance of these residues. We found the highly conserved amino acid residues at both phosphor-site and β5 sheet provided the most beneficial energy contributions for both phosphorylated peptide and unphosphorylated peptide. The results also implied the binding interaction of different ligands among DLGs should be the similar. The slightly difference only came from the selective sites outside the GMP-binding subdomain. Therefore, we inferred the DLG4 synthesized peptide binder, GKI-QSF, could also bind with DLG1. After analyzing the molecular dynamic results, we predicted GKI-QSF might bind with DLG1 with similar binding affinity, which was further confirmed by using ITC with *K*d value of 1.20 ± 0.29 μM. These results of theoretical predication and experimental verification indicated our study might be helpful for the better understanding of the biological function of DLGs and will encourage the discovery of new binder in the future.

## Data Availability Statement

All datasets generated and analyzed for this study are included in the article/Supplementary Material.

## Author Contributions

All authors listed have made a substantial, direct and intellectual contribution to the work, and approved it for publication.

### Conflict of Interest

The authors declare that the research was conducted in the absence of any commercial or financial relationships that could be construed as a potential conflict of interest.
