# Supplementary material for: Characterizing the Binding Sites for GK Domain of DLG1 and DLG4 via Molecular Dynamics Simulation
Source: Front Mol Biosci. 2020 Jan 23;7:1. doi: 10.3389/fmolb.2020.00001 (PMC6989407; doi:10.3389/fmolb.2020.00001)
Supplement: Supplementary file 1 [file Data_Sheet_1.PDF]

Supplementary Material

Characterizing the Binding Sites for GK domain of DLG1 and DLG4 *via* Molecular Dynamics Simulation

Hongwei Li[1#], Qiong Chen[2#], Changyu Shan[1], Chunling Guo[1], Xiuming Yang[1], Yingchun Chen[1], Jinwei Zhu[3], Qin Ouyang[1*]

[1] Department of Pharmaceutical Chemistry, Third Military Medical University, Chongqing, China

[2] Department of Neurology, Xinqiao Hospital, Third Military Medical University, Chongqing, China

[3] Bio-X Institutes, Key Laboratory for the Genetics of Developmental and Neuropsychiatric Disorders, Ministry of Education, Shanghai Jiao Tong University, Shanghai, China

Email: ouyangq@tmmu.edu.cn

1. Supplementary Figures

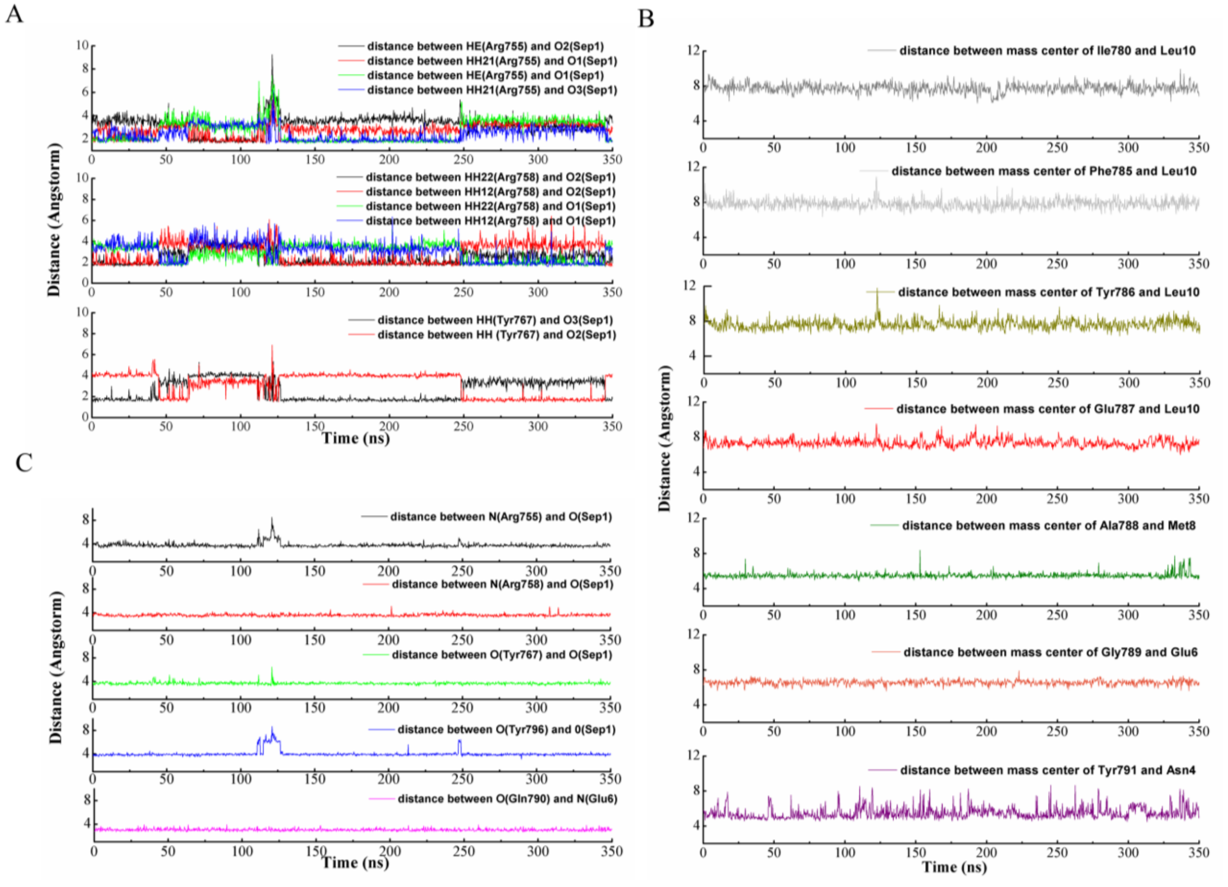

Supplementary Figure 1 (A) The distances between H atom of the residues (Arg755, Arg788 and Tyr767) and O atom of the residue p-Sep1 in p-LGN. **(B)** The distances between the mass centers of the residues possessing the hydrophobic interactions in DLG1/p-LGN complex. **(C)** The distances between the N/O atom of the residues in DLG1 and O/N atom of the residues in p-LGN that formed the hydrogen bonds.

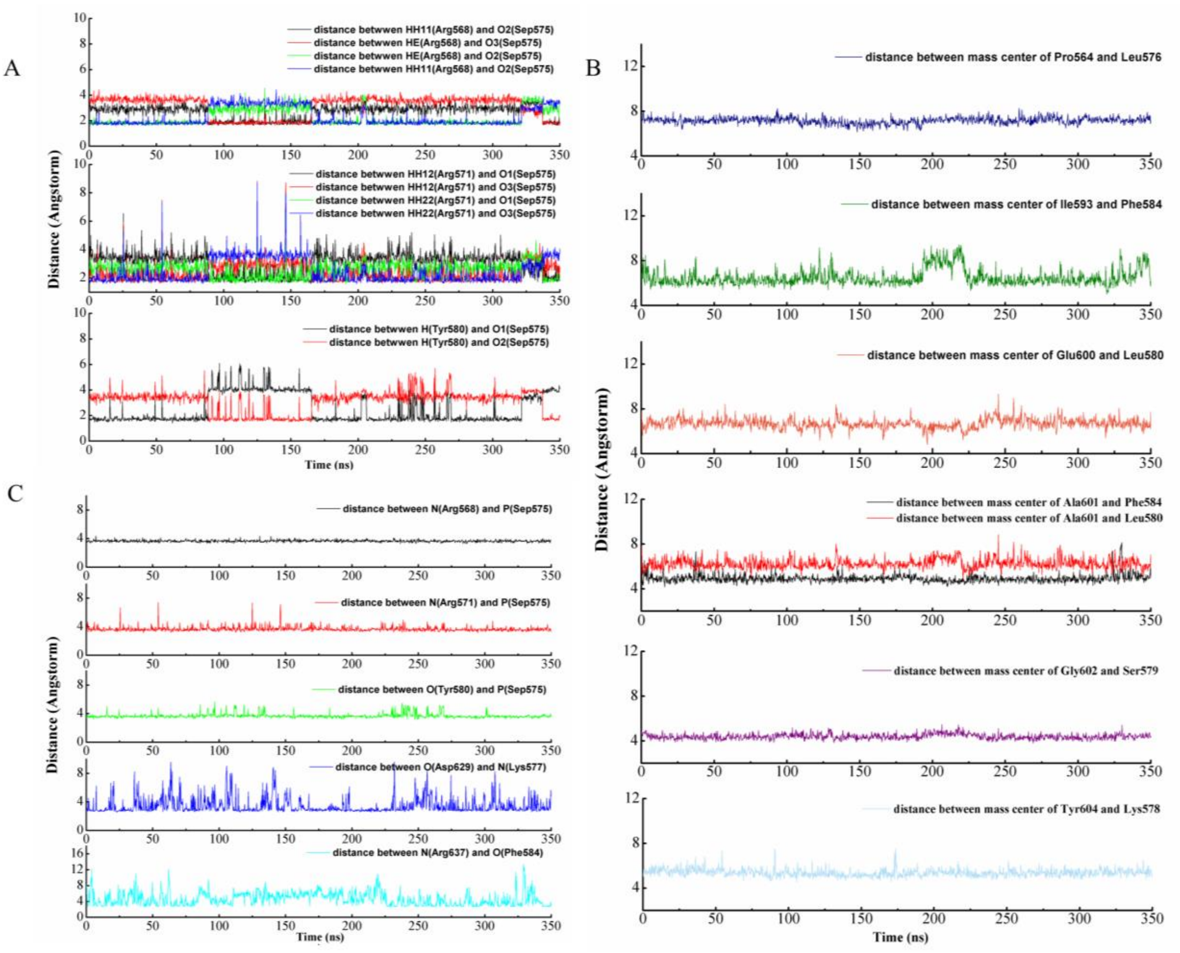

Supplementary Figure 2 (A) The distances between H atom of the residues (Arg568, Arg571 and Tyr580) and O atom of the residue p-Sep575 in p-LGL2. **(B)** The distances between the mass centers of the residues possessing the hydrophobic interactions in DLG4/p-LGL2 complex. **(C)** The distances between the N/O atom of the residues in DLG4 and O/N atom of the residues in p-LGL2 that formed the hydrogen bonds.

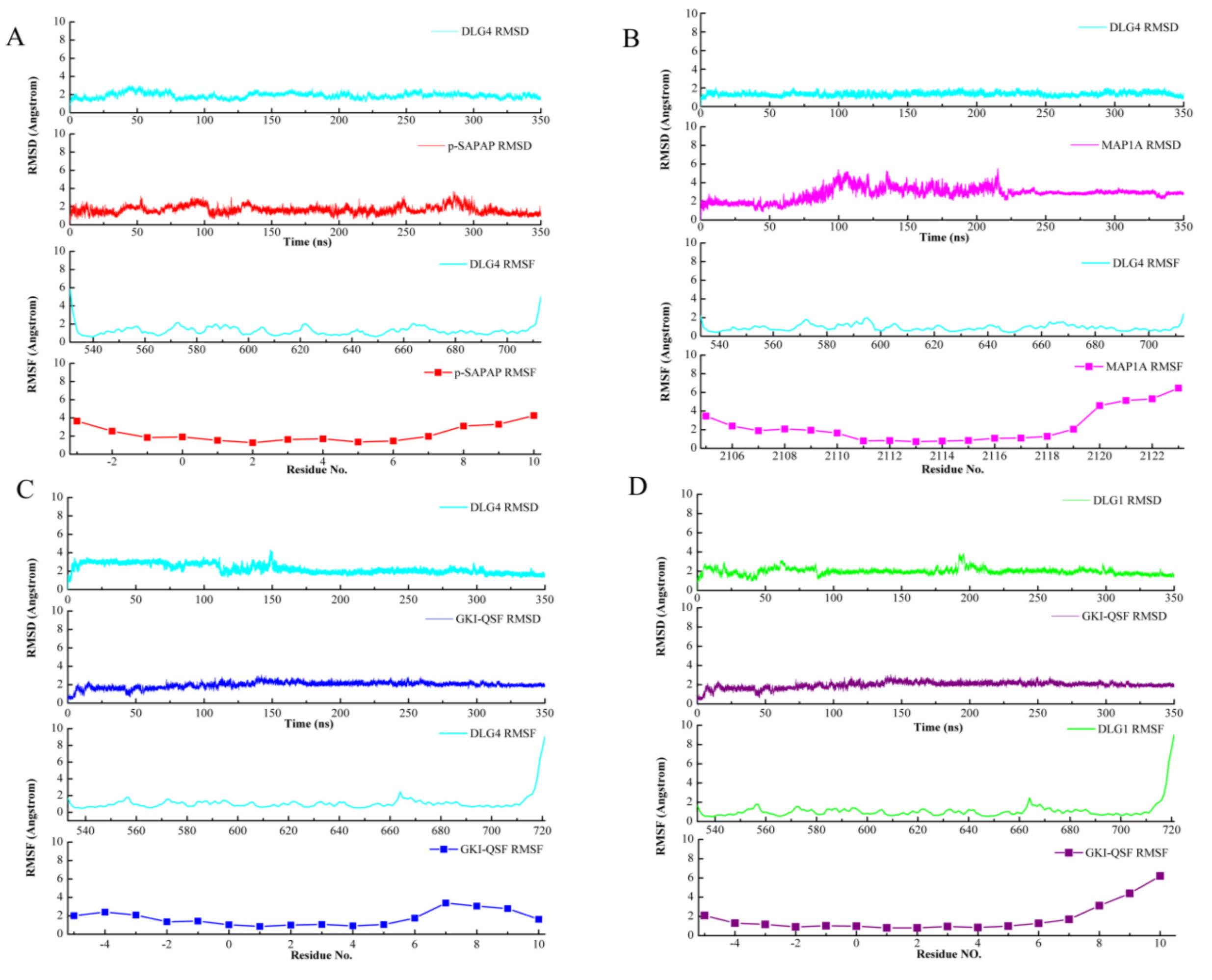

Supplementary Figure 3 (A) The RMSD and RMSF of the DLG4 and p-SAPAP. **(B)** The RMSD and RMSF of the DLG4 and MAP1A. **(C)** The RMSD and RMSF of the DLG4 and GKI-QSF. **(D)** The RMSD and RMSF of the DLG1 and GKI-QSF.

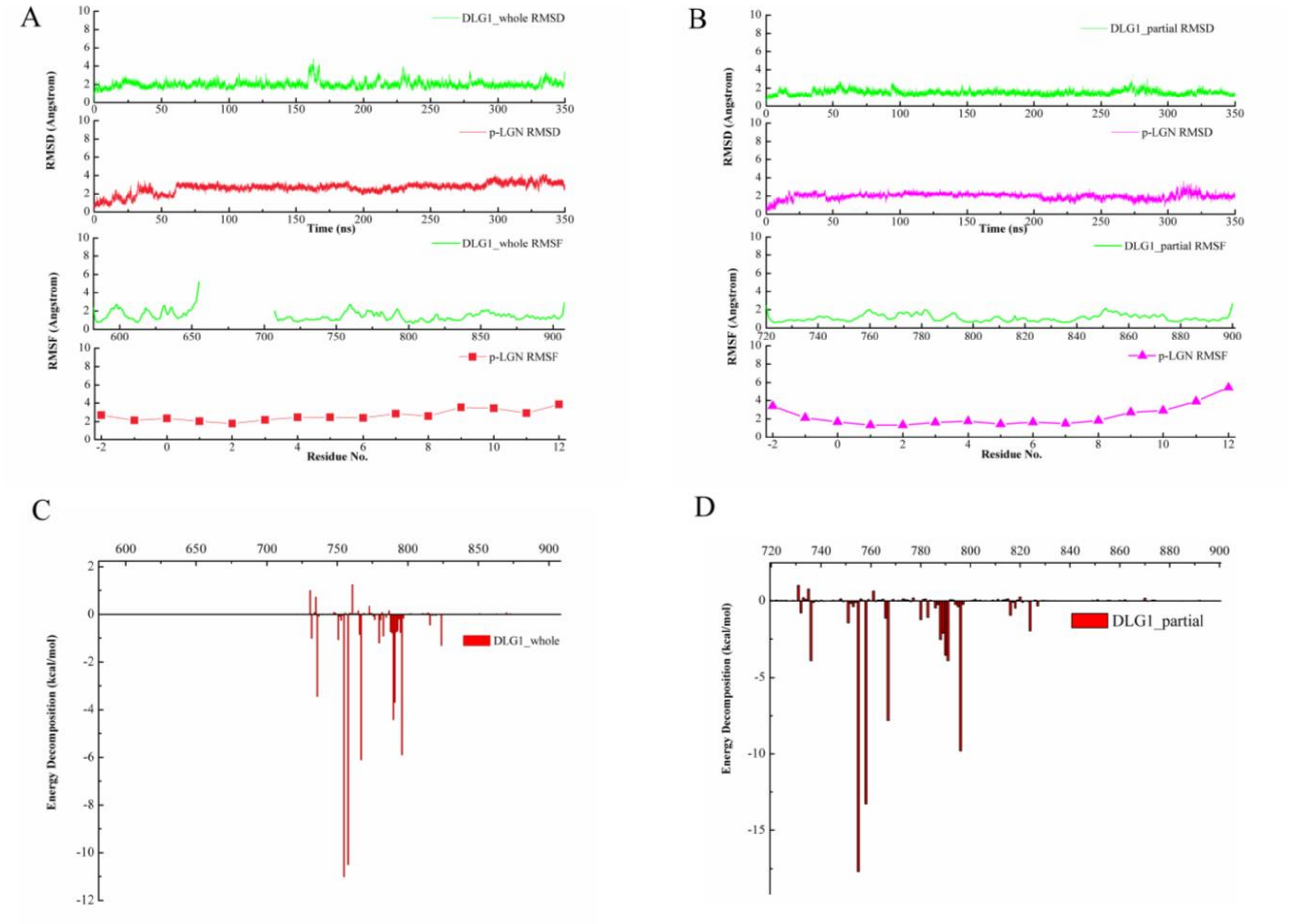

Supplementary Figure 4 (**A**) The RMSD and RMSF of DLG1_whole (the whole DLG1 crystal structures) and p-LGN. **(B)** The RMSD and RMSF of DLG1_partial (the partial structures that deleted the redundant amino acids) and p-LGN. The RMSD values of DLG1_partial system dynamic simulations were very similar as DLG1_whole system which were stable at 2 Å. For the RMSF, the DLG1_whole and DLG1_partial had the same distributions, while the loop regions at N- and C-terminus of the ligand p-LGN in DLG1_partial system had a relatively larger values than DLG1_whole. **(C, D)** Binding free energy decomposition of DLG1_whole and DLG1_partial systems. The two systems had the similar energy distributions.

2. Supplementary Tables

Supplementary Table 1. The binding free energy decomposition of the residues greater than 1 kcal/mol in DLG1/p-LGN

Residue	van der Waals	Electrostatic	Polar Solvation	Non-Polar Solv.	TOTAL
ARG755	0.44 ± 0.78	-60.10 ± 5.79	41.19 ± 4.99	-0.01 ± 0.01	-18.48 ± 1.86
ARG758	0.70 ± 0.97	-56.94 ± 7.73	42.10 ± 6.42	-0.18 ± 0.04	-14.33 ± 2.67
TYR796	-2.1 ± 1.01	-11.18 ± 1.51	3.89 ± 0.91	-0.30 ± 0.05	-9.69 ± 1.02
TYR767	-0.40 ± 0.92	-15.18 ± 1.65	8.33 ± 0.91	-0.30 ± 0.06	-7.54 ± 0.83
TYR791	-4.29 ± 0.80	-2.00 ± 1.52	3.19 ± 1.38	-0.61 ± 0.10	-3.71 ± 0.89
GLN790	-2.77 ± 0.60	-3.90 ± 2.93	3.56 ± 2.13	-0.41 ± 0.07	-3.52 ± 1.11
ALA788	-2.51 ± 0.42	-3.11 ± 0.59	3.23 ± 0.41	-0.36 ± 0.03	-2.75 ± 0.55
ASP816	-0.61 ± 0.91	-35.01 ± 19.01	33.40 ± 16.56	-0.46 ± 0.11	-2.67 ± 2.82
ASP736	0.26 ± 0.87	-33.27 ± 19.07	30.60 ± 15.95	-0.15 ± 0.11	-2.56 ± 3.22
GLY789	-2.56 ± 0.32	-3.27 ± 0.62	3.98 ± 0.42	-0.28 ± 0.02	-2.14 ± 0.40
HIP783	-0.92 ± 0.69	-31.05 ± 10.73	30.73 ± 9.73	-0.37 ± 0.17	-1.61 ± 1.43
ARG824	-2.46 ± 0.59	-19.96 ± 4.64	21.32 ± 4.31	-0.49 ± 0.08	-1.59 ± 1.10
ASP732	-0.91 ± 0.90	-9.20 ± 14.81	8.95 ± 12.78	-0.23 ± 0.10	-1.39 ± 2.15
PRO751	-1.06 ± 0.32	0.15 ± 0.61	-0.26 ± 0.60	-0.20 ± 0.06	-1.37 ± 0.46
ILE780	-1.18 ± 0.29	0.84 ± 0.45	-0.64 ± 0.46	-0.27 ± 0.04	-1.26 ± 0.30

All the energies are in kcal/mol.

Supplementary Table 2. The binding free energy decomposition of the residues greater than 1 kcal/mol in DLG4/p-LGL2

Residue	van der Waals	Electrostatic	Polar Solvation	Non-Polar Solv.	TOTAL
ARG568	0.56 ± 0.97	-10.49 ± 7.34	-7.91 ± 6.04	-0.01 ± 0.01	-17.85 ± 1.81
ARG571	0.34 ± 0.78	-0.16 ± 9.26	-12.51 ± 8.13	-0.23 ± 0.07	-12.56 ± 2.13
TYR609	-2.97 ± 0.89	-9.56 ± 1.64	2.42 ± 0.93	-0.30 ± 0.03	-10.41 ± 0.82
TYR580	-0.85 ± 1.06	-12.99 ± 2.69	7.16 ± 1.22	-0.32 ± 0.06	-7.00 ± 1.39
ARG637	0.18 ± 0.86	-18.33 ± 9.85	12.69 ± 7.02	-0.27 ± 0.09	-5.74 ± 2.80
TYR604	-4.91 ± 0.62	-0.61 ± 2.68	1.75 ± 2.22	-0.71 ± 0.08	-4.47 ± 0.86
GLY602	-2.83 ± 0.46	-1.63 ± 1.06	1.93 ± 0.71	-0.29 ± 0.03	-2.82 ± 0.76
GLN603	-3.47 ± 0.85	-4.58 ± 2.58	5.92 ± 1.91	-0.51 ± 0.09	-2.64 ± 1.23
ALA601	-2.13 ± 0.57	-1.29 ± 1.78	1.71 ± 1.10	-0.30 ± 0.04	-2.00 ± 0.90
ILE593	-1.12 ± 0.33	0.49 ± 0.43	-0.45 ± 0.41	-0.23 ± 0.05	-1.31 ± 0.37
ASP629	-0.17 ± 0.68	-76.95 ± 11.54	76.12 ± 10.73	-0.31 ± 0.08	-1.31 ± 1.28

All the energies are in kcal/mol.

Supplementary Table 3. The reported crystal structures of DLGs GK domain with the binding parteners

PDB ID	DLGs	Binding parteners	Descriptions	References
3W9Y	DLG1 /SAP97	Another C-terminal tail of DLG1	DLG1 /SAP97	Mori et al., 2013
3UAT	DLG1 /SAP97	p-LGN	the mitotic spindle regulatory protein	Zhu et al., 2011
3WP0	DLG4/PSD95	p-LGL2	Lethal giant larvae	Zhu et al., 2014
3WP1	DLG4/PSD95	p-LGL	Lethal giant larvae	Zhu et al., 2014
5YPO	DLG4/PSD95	p-SAPAP	SAP90/PSD-95-associated protein	Zhu et al., 2017
5YPR	DLG4/PSD95	GKI-QSF	Synthesized_peptide	Zhu et al., 2017
5B64	DLG4/PSD95	Kif13b	Kinesin-like protein	Zhu et al., 2016
5GNV	DLG4/PSD95	MAP1A	Microtubule-associated protein	Xia et al., 2017

Reference:

Mori, S., Tezuka, Y., Arakawa, A., Handa, N., Shirouzu, M., Akiyama, T., et al. (2013). Crystal structure of the guanylate kinase domain from discs large homolog 1 (DLG1/SAP97). *Biochem. Biophys. Res. Commun*. 435(3), 334-338. doi: 10.1016/j.bbrc.2013.04.056.

Zhu, J., Shang, Y., Xia, C., Wang, W., Wen, W., and Zhang, M. (2011). Guanylate kinase domains of the MAGUK family scaffold proteins as specific phospho-protein-binding modules. *EMBO J* 30(24)**,** 4986-4997. doi: 10.1038/emboj.2011.428.

Zhu, J., Shang, Y., Wan, Q., Xia, Y., Chen, J., Du, Q., et al. (2014). Phosphorylation-dependent interaction between tumor suppressors Dlg and Lgl. *Cell Res* 24(4)**,** 451-463. doi: 10.1038/cr.2014.16.

Zhu, J., Zhou, Q., Shang, Y., Li, H., Peng, M., Ke, X., et al. (2017). Synaptic Targeting and Function of SAPAPs Mediated by Phosphorylation-Dependent Binding to PSD-95 MAGUKs. *Cell Rep* 21(13)**,** 3781-3793. doi: 10.1016/j.celrep.2017.11.107.

Zhu, J., Shang, Y., Xia Y, Zhang, R., Zhang, M. (2016) An Atypical MAGUK GK Target Recognition Mode Revealed by the Interaction between DLG and KIF13B. *Structure* 24(11),1876-1885. doi: 10.1016/j.str.2016.08.008.

Xia, Y., Shang, Y., Zhang, R., and Zhu, J. (2017). Structure of the PSD-95/MAP1A complex reveals a unique target recognition mode of the MAGUK GK domain. *Biochem J* 474(16)**,** 2817-2828. doi: 10.1042/BCJ20170356.
